# Clicking in Shallow Rivers: Short-Range Echolocation of Irrawaddy and Ganges River Dolphins in a Shallow, Acoustically Complex Habitat

**DOI:** 10.1371/journal.pone.0059284

**Published:** 2013-04-03

**Authors:** Frants H. Jensen, Alice Rocco, Rubaiyat M. Mansur, Brian D. Smith, Vincent M. Janik, Peter T. Madsen

**Affiliations:** 1 Department of Biology, Woods Hole Oceanographic Institution, Woods Hole, Massachusetts, United States of America; 2 Wildlife Conservation Society, Bangladesh Cetacean Diversity Project, Shonadanga R/A, Khulna, Bangladesh; 3 Wildlife Conservation Society, Asian Freshwater and Coastal Cetacean Program, Bronx, New York, United States of America; 4 Sea Mammal Research Unit, School of Biology, University of St. Andrews, St. Andrews, Fife, United Kingdom; 5 Zoophysiology, Department of Bioscience, Aarhus University, Aarhus, Denmark; Texas A&M University-Corpus Christi, United States of America

## Abstract

Toothed whales (*Cetacea, odontoceti*) use biosonar to navigate their environment and to find and catch prey. All studied toothed whale species have evolved highly directional, high-amplitude ultrasonic clicks suited for long-range echolocation of prey in open water. Little is known about the biosonar signals of toothed whale species inhabiting freshwater habitats such as endangered river dolphins. To address the evolutionary pressures shaping the echolocation signal parameters of non-marine toothed whales, we investigated the biosonar source parameters of Ganges river dolphins (*Platanista gangetica gangetica*) and Irrawaddy dolphins (*Orcaella brevirostris*) within the river systems of the Sundarban mangrove forest. Both Ganges and Irrawaddy dolphins produced echolocation clicks with a high repetition rate and low source level compared to marine species. Irrawaddy dolphins, inhabiting coastal and riverine habitats, produced a mean source level of 195 dB (max 203 dB) re 1 µPa_pp_ whereas Ganges river dolphins, living exclusively upriver, produced a mean source level of 184 dB (max 191) re 1 µPa_pp_. These source levels are 1–2 orders of magnitude lower than those of similar sized marine delphinids and may reflect an adaptation to a shallow, acoustically complex freshwater habitat with high reverberation and acoustic clutter. The centroid frequency of Ganges river dolphin clicks are an octave lower than predicted from scaling, but with an estimated beamwidth comparable to that of porpoises. The unique bony maxillary crests found in the Platanista forehead may help achieve a higher directionality than expected using clicks nearly an octave lower than similar sized odontocetes.

## Introduction

Bats and toothed whales have independently evolved a sophisticated biosonar system [1], [2], allowing both clades to diversify and occupy many different niches [3], [4]. Toothed whales constitute a morphologically and ecologically diverse group of predators, inhabiting every ocean and several large, freshwater river systems [5]. Some species forage on deep-sea squid at mesopelagic depths (e.g. sperm whales [6], [7]), others prey on large schools of fish sparsely distributed in oceanic habitats (e.g. dusky dolphins [8]) or on individual shrimp and fish encountered in shallow river systems inhabited by several species of river dolphins, including Irrawaddy and Ganges river dolphins [9]. While the biosonar signals of many marine toothed whales have been studied in detail [10], [11], [12], we know little about the polyphyletic assembly of true river dolphins and how the biosonar of these old lineages have evolved to their freshwater habitat [13].

Toothed whale biosonar signals have been studied in captivity over the last 60 years and increasingly also in the wild. Studies of captive animals have contributed greatly towards our understanding of the biosonar performance [14] including dynamic biosonar control [15], [16]. Studies of free-ranging animals complement laboratory studies by revealing how animals use echolocation in the wild, where the natural habitat may have physical characteristics very different from captive settings [12]. Four different types of odontocete biosonar signals have been identified: Sperm whales produce highly directional echolocation signals characterized by low centroid frequency and very high peak-to-peak source level (SL) exceeding 235 dB_pp_ re 1 µPa @1 m [17], [18], which enables them to echolocate deep-sea squid or other prey at relatively long range [19]. Whistling delphinids use very short, broadband clicks with centroid frequencies above 60–80 kHz [12], [20], [21], [22], [23] and peak-to-peak SL of 210–228 dB [12]. Beaked whales produce frequency-modulated clicks centered around 45 kHz [24], [25], [26]. Peak-to-peak source levels are slightly lower than delphinid clicks, but due to their much longer duration, they contain comparable amounts of energy [25], [26]. Lastly, a polyphyletic assemblage of porpoises, six non-whistling delphinids of the Cephalorhynchus and Lagenorhynchus families, pygmy sperm whales (*Kogia sp.*), and the Franciscana dolphin (*Pontoporia franciscana*) all use Narrow Band High Frequency (NBHF) clicks where energy is concentrated in a narrow frequency band around 130 kHz [27]. These animals seem to produce nearly as directional biosonar signals as delphinids [15], [27], [28] but at lower source levels [27], [28], [29].

Despite the many studies quantifying sonar parameters for free-living, marine toothed whales, much less variation in signal type or biosonar parameters has been found compared to bats, especially among delphinids. However, most of the delphinids studied to date forage in habitats that may differ less acoustically than is the case for the different bat guilds. Instead it seems that an inverse scaling of frequency with body mass to achieve a similar directionality may be a major driving force across the toothed whale suborder [15]. However, it is unclear how these selective pressures for high amplitude, high source level biosonar signals can be extrapolated to the acoustically complex, relatively shallow and turbid environments inhabited by river dolphins.

**Figure 1:**
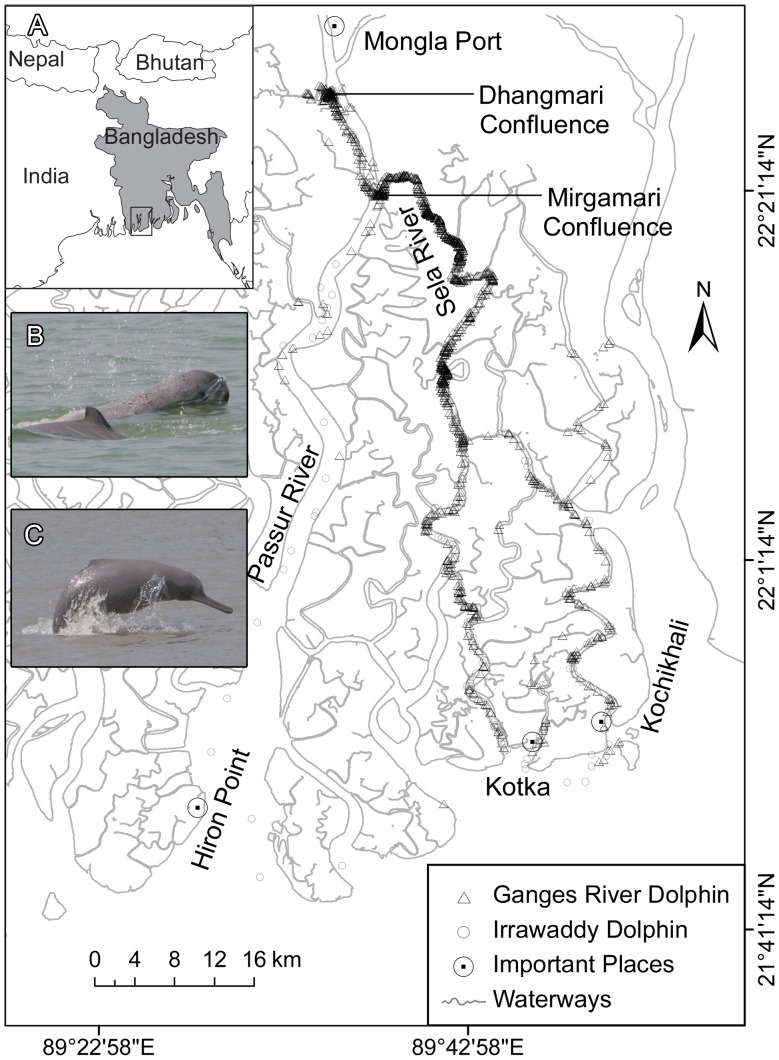
Field site and distribution of Irrawaddy dolphins and Ganges river dolphins. A) Map of the Sundarbans mangrove forest, Bangladesh, including sighting data of Ganges river dolphins (triangles) and Irrawaddy dolphins (circles). Adapted with permission from Smith et al. [73]. Inserts show pictures of B) Irrawaddy dolphin, and C) Ganges river dolphin, taken by E. & R. Mansur, WCS.

To address this question, we studied two species of toothed whales that co-occur in waterways of the Sundarbans mangrove forest of Bangladesh (Fig. 1). Irrawaddy dolphins (*Orcaella brevirostris*) are freshwater cetaceans living in shallow coastal waters, generally associated with freshwater inputs, as well as far upstream in three large, Indo-Pacific river systems. The extent of their inland range in the Sundarbans varies with seasonal freshwater regimes [30] and may be influenced by the distribution of Ganges river dolphins [31]. Ganges river dolphins (*Platanista gangetica gangetica*) are obligate freshwater dolphins found in the Ganges, Brahmaputra and Karnaphuli river systems where they exhibit a peculiar, side-swimming form of locomotion [32]. The extent of their downstream range in the Sundarbans is also determined by seasonally dynamic freshwater flows [30], with the Ganges river dolphin favouring low salinity, high turbidity and moderate depth [33]. Both Irrawaddy dolphins (Fig. 1B) and Ganges river dolphins (Fig. 1C) have relatively small bodies comparable to small marine delphinids and porpoises [9]. In the Sundarbans, they inhabit geomorphically complex areas with extremely variable depth, salinity and turbidity in contrast to the more stable characteristics of marine environments [33]. Given the complex acoustic environment and high amount of clutter and reverberation, it may be hypothesized that Irrawaddy dolphins and Ganges river dolphins employ echolocation signals characterized by low-amplitude, high frequency sonar signals emitted at high repetition rates like small bat species hunting in cluttered habitats [34].

In this study, we quantify the biosonar source parameters of Ganges river dolphins and Irrawaddy dolphins to test this hypothesis. We show that these animals use consistently lower source levels and higher repetition rates than oceanic delphinids, possibly limited by high amounts of clutter and reverberation. We demonstrate that Ganges river dolphins have a slightly broader beamwidth than other toothed whales due to their very low centroid frequency but that they achieve a higher directionality than expected from a direct scaling with centroid frequency and size, possibly by using a novel set of bony plates in the forehead. We conclude this study by discussing means to use acoustics to help better understand the conservation needs of these highly endangered freshwater toothed whales.

## Materials and Methods

### Study Area

Recordings were obtained in the waterways of the Bangladesh part of the Sundarban mangrove forest (Fig. 1) where recording depths varied from 6.5 to 23 m, (mean 12.94 m). Recordings took place during daylight hours between the 4^th^ and16^th^ of February 2010 from a 12 m long, wooden research boat. All research was conducted under a research permit issued to the Bangladesh Cetacean Diversity Project of the Wildlife Conservation Society by the Ministry of Environment and Forest, Government of Bangladesh.

### Recording Equipment

A vertical array of four Reson TC4034 spherical hydrophones (Reson A/S, Slangerup, Denmark) was formed by mounting hydrophones in a Perspex rod (4 cm diameter, hollow) with 0.75 m spacing. The first hydrophone was positioned at 2 m depth while the last hydrophone was at 4.25 m depth. A buoy was attached to the top of the array, and a 4 kg weight was fixed to the bottom to help maintain the array vertical in the water. Signals were amplified 60 dB by a custom-made amplifier and filter box (1 kHz 1-pole high-pass and 200 kHz 4-pole low-pass filter), then digitized by two synchronized National Instruments USB-6251 A/D converters (National Instruments, Texas, USA) at a sampling rate of 500 kHz per channel and a resolution of 16 bits. The calibrated clip level of the recording chain was 174****dB re µPa (peak), and the frequency response of the recording chain was flat (±2 dB) from 2–180 kHz.

### Data Collection

Ganges river dolphins were recorded while foraging or resting at the convergences of channels. Irrawaddy dolphins were recorded during different behaviors (travelling, foraging, and socializing). The boat engine was turned off and the array was lowered into the water once the animals were within about 100 m of the vessel. Data acquisition was initiated and terminated manually and files were stored approximately every minute. Start and end time, position and depth were recorded for every recording event, as well as group composition and behavior.

### Click Analysis

Signal analysis was carried out with custom-written routines in Matlab 7.5 (The Mathworks, Inc., Natwick, MA, USA). Each click series (also referred to in the literature as a click train) was examined visually and discarded if more than one animal was present to avoid underestimating interclick intervals. Echolocation clicks were then located on the third hydrophone using an automated click detector with a variable detection threshold chosen during visual inspection of waveforms to exceed the background noise level and detect individual click series. Each click was further analyzed only if detected on all four channels.

### Acoustic Localization

Source location relative to the hydrophones was obtained through acoustic localization techniques based on time-of-arrival differences of the same click on the four receivers [35], [36]. To find the time of arrival differences, the signal recorded on the top hydrophone was cross-correlated with the signals recorded on the other hydrophones, excluding surface reflections. A sound speed of 1500 m/s was measured in each recording habitat by emitting pulses with a portable echosounder (Speedtech, Virginia, USA) at the position of the top hydrophone and cross-correlating to find the time-of-arrival at the remaining hydrophones at known distances. For each pair of hydrophones, the time-of-arrival difference can be explained by the equation for a single hyperbola in the two-dimensional plane of the array. Using four receivers, equations for three independent hyperbolas can be generated, and the position of the sound source found by solving the three equations with a least-squares method [35], [37].

Acoustic localization with this array was calibrated in Aarhus Harbour, Denmark, using artificial clicks (2 cycles at 70 kHz) generated by an omnidirectional HS70 hydrophone (Sonar Products) connected to a waveform generator (model 33220A, Agilent Technologies, California, USA). Pulses were emitted from a depth of 2 m and at distances from 5 m to 40 m from the array. Speed of sound during this calibration was calculated using the Leroy equation [38] from measured temperature and salinity values.

### Source Parameter Estimation

The interclick interval (ICI) was defined as the time between each click and the previous [14]. Received levels were calculated as peak-peak (pp) and root-mean-square (rms) sound pressure levels [14] within a time window given by the −10 dB end points relative to the peak of the amplitude envelope [39]. The temporal duration of clicks was defined as the length of the −10 dB time window. The energy flux density was calculated for each click as the sum of squared sound pressure values within the −10 dB analysis window [39]. Subsequently, the click power spectrum was calculated as the squared Fast Fourier Transform of a 32-point window centred on the peak envelope of each signal. The power spectrum was then normalized and interpolated with a factor of 100 using a low-pass interpolation. Peak frequency, centroid frequency (defined as the frequency separating the power spectrum into two halves of equal energy) and signal bandwidth (−3 dB power and −10 dB power) was calculated from this power spectrum [40]. Source levels (SL) were defined as the back-calculated sound pressure level 1 m from the source on the acoustic axis [37], [41] and calculated from received levels by compensating for the transmission loss (dB re. 1 m), estimated as the combination of spherical spreading and frequency-dependent absorption (taken at the centroid frequency of the received click) over the range from the source coordinates to the receiver.

### On-axis Criteria

Off-axis signals are subjected to distortion [11], [12], [14]. This means that it is essential to quantify the signal on or as close as possible to the acoustic axis when investigating source parameters of highly directional biosonar signals [37]. With a linear array, the vertical angle of incidence can be estimated, but the horizontal angle of incidence is unknown. To maximize the likelihood of analyzing on-axis clicks, we selected only the highest-amplitude click in a longer click sequences (scans) with clicks of increasing and decreasing amplitude. These scans are most likely associated with the acoustic beam of the animal passing across the axis of the array [18]. Assuming the animal maintains the same source level and directionality, the click with the highest amplitude has the highest likelihood of being on-axis in the horizontal plane [23]. The criteria used to determine if the click was on axis is similar to that described in previous studies with similar arrays [11], [23], [27], [28]: (1) the click could be localized; (2) the click had the highest received level in a scan (and thus assumed to be on-axis in the horizontal plane); and (3) the highest received level was recorded on one of the two central hydrophones, allowing for estimation of the angle of incidence in the vertical plane.

### Implications for Passive Acoustic Monitoring

To evaluate the use of sound source parameters for passive acoustic monitoring studies without the potential for identifying on-axis clicks, a set of click series with only one clicking animal was identified. Each of these click series was passed through an automatic click detector (described above) to find accurate inter-click intervals for the two species. Subsequently, the power spectrum of each click was analyzed to find the centroid frequency.

## Results

Irrawaddy dolphins were recorded on 16 different occasions during a total of 9 hours, 58 minutes of recordings. The median group size encountered during recordings of Irrawaddy dolphins was 3 animals. During recordings, this species was observed while foraging and travelling. Ganges river dolphins (median group size 4 animals) were recorded in two different occasions and a total of 57 minutes of recordings were obtained from these encounters. In both recording occasions, the Ganges river dolphins were located in channel convergences.

**Figure 2:**
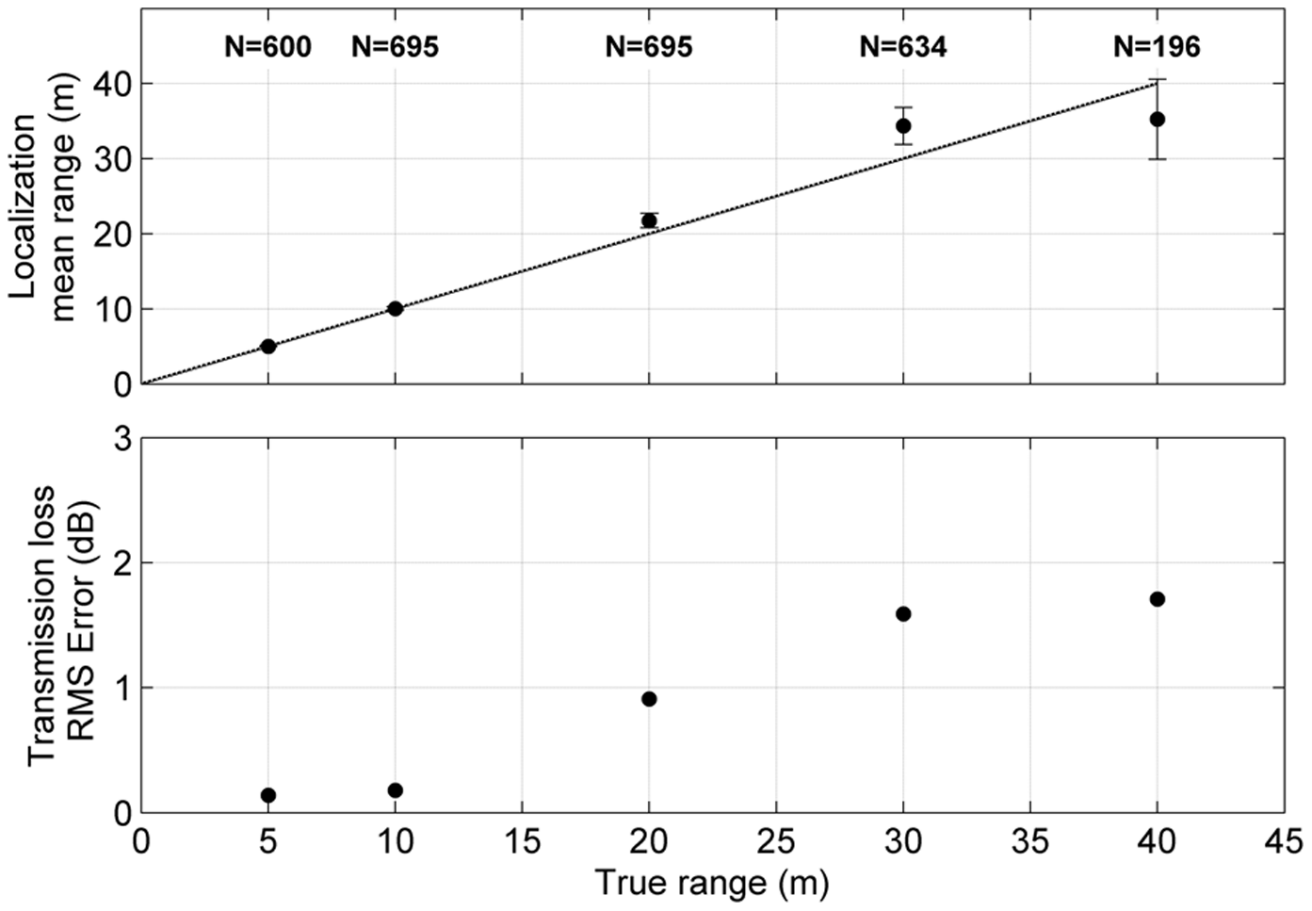
Calibration of the acoustic localization procedure with a vertical 4-hydrophone array. Top: Localization range (mean ± SD) given by the acoustic localization procedure, as a function of the calibration distance. Precise localization indicated by the dotted line. Bottom: RMS error in the estimated transmission loss as a function of range from the array.

The hydrophone localization calibration indicated that clicks within 40 m were localized with a resulting error in the transmission loss estimates of less than 3 dB (Fig. 2), which was deemed acceptable in accordance with previous studies [23], [27], [28]. Consequently, only clicks recorded within a 40 m range of the hydrophone array were used for the analysis of the source parameters.

**Figure 3:**
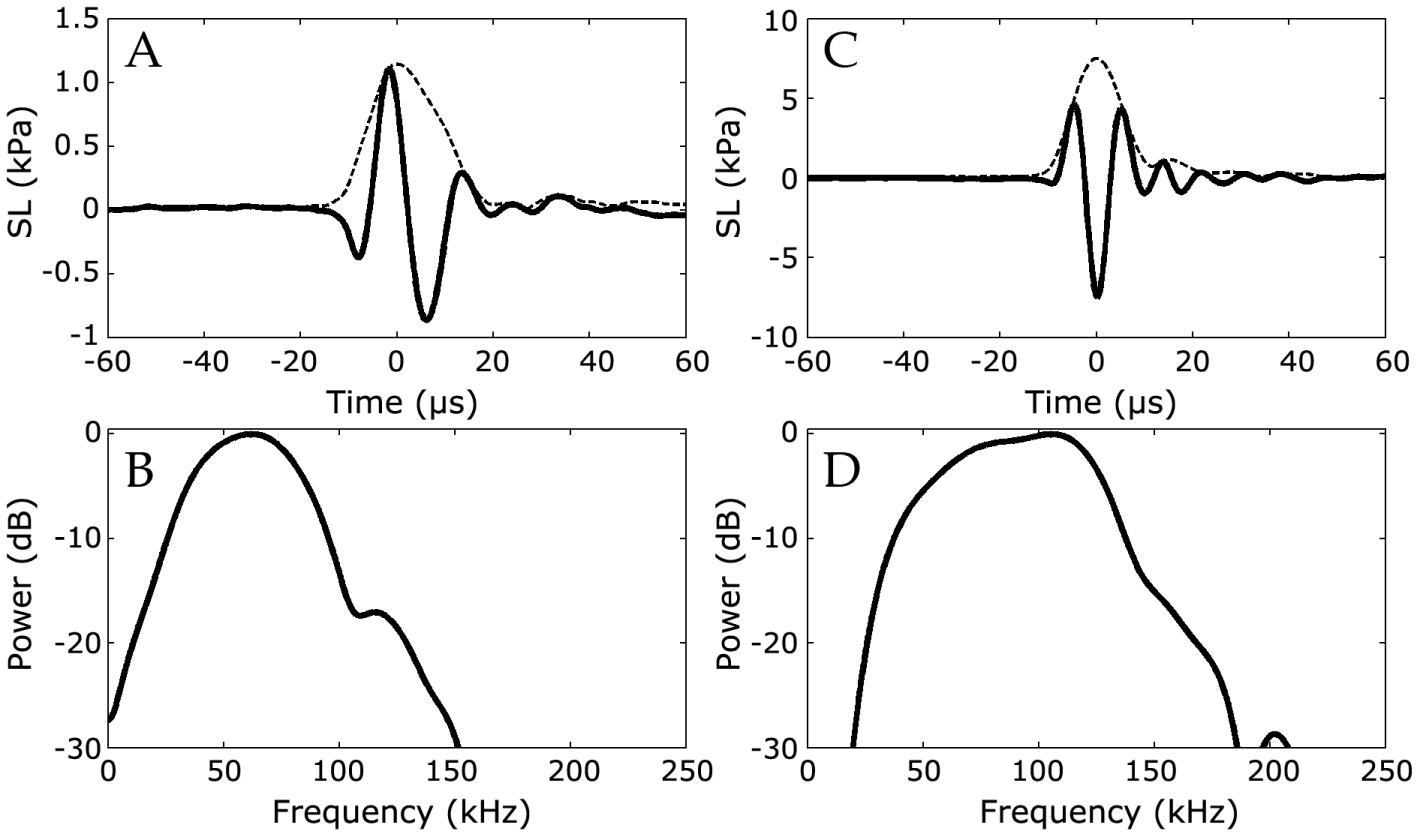
Representative echolocation clicks from Ganges river dolphins and Irrawaddy dolphins. A: Signal waveform (solid line) and envelope (interrupted line) of a Ganges river dolphin echolocation click. B: Normalized power spectrum of a Ganges river dolphin echolocation click. C: Signal waveform (solid line) and envelope (interrupted line) of Irrawaddy dolphin echolocation click. D: Normalized power spectrum of Irrawaddy dolphin echolocation click. Time-domain signal is shown as the instantaneous source level, corrected for transmission loss and absorption between source position and hydrophone (note the different amplitude scales). Power spectra are constructed from a 32-point rectangular window around the peak of the envelope, and interpolated with a factor 320, for a spectral resolution of 24 Hz.

A total of 15 Irrawaddy dolphin and 29 Ganges river dolphin clicks met the on-axis criteria and were recorded within the localization range of 40 meters. Only one click from each scan was used for analysis, and all recording areas were well separated to prevent recording the same groups of animals repeatedly. Clicks for both species were broadband transients (Fig. 3) similar to those of marine, whistling delphinids [14], [25]. Mean click duration ± SD was 13.4±3.0 µs for Irrawaddy dolphins and 21.7±2.2 µs for Ganges river dolphins, and Q ratios (defined as the ratio of centroid frequency to RMS bandwidth) was 3.2±0.3 (mean±SD) for Irrawaddy dolphins and 3.1±0.3 for Ganges river dolphins.

**Figure 4:**
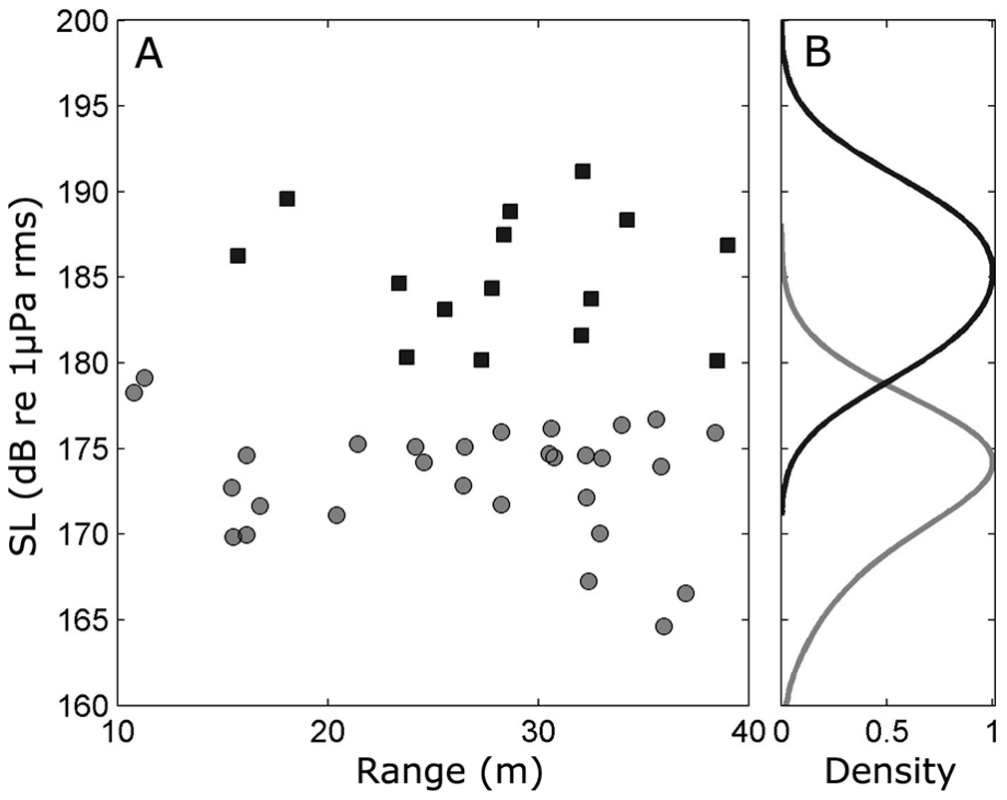
Source levels of Irrawaddy dolphins and Ganges river dolphins. A) Estimated RMS source levels (SL) as a function of range between hydrophone array and estimated source position for both Irrawaddy dolphins (black) and Ganges river dolphins (grey). B) Normalized density estimates of the SL from both species, estimated using normal kernels with a 3 dB kernel width.

Ganges river dolphin click source levels were significantly lower than the source levels of Irrawaddy dolphin clicks (Kruskal-Wallis: p<0.0001) (Table 1). Peak-to-peak source levels (mean±SD) were 194.5±3.6 dB re 1 µPa at 1 m for Irrawaddy dolphins and 183.3±3.4 dB re 1 µPa at 1 m for Ganges river dolphins. For both species, these source levels are significantly lower (Kruskal-Wallis: p<0.0001) than source levels produced by a marine delphinid, the Indopacific Bottlenose dolphin (*Tursiops aduncus*) recorded in a 5–8 m shallow bay (mean peak-to-peak source levels ± SD of 205±7 dB re 1 µPa at 1 m [12], [23]) and lower than published source levels from most other free-ranging toothed whales with the exception of some species producing narrow-band high-frequency clicks (Table 2). Similarly, the mean source energy flux density was 136.3 dB re 1 µPa^2^*s at 1 m for Irrawaddy dolphins and 126.6 dB re 1 µPa^2^*s at 1 m for Ganges river dolphins. There was no significant relationship between the recording range and the source levels for either species (Kruskal-Wallis: p = 0.46 for Ganges river dolphins and p = 0.45 for Irrawaddy dolphins) (Fig. 4). The centroid frequency (mean±SD) for Irrawaddy dolphins was 94.6±9.7 kHz, with −3 dB bandwidth of 64.4±15.8 kHz. Ganges river dolphins had a significantly lower centroid frequency (mean±SD) of 61.4±4.9 kHz (Kruskal-Wallis: p<0.001) and correspondingly also a significantly lower −3 dB bandwidth of 43.8±7.1 dB (Kruskal-Wallis: p<0.001).

**Table 1:** Biosonar parameters of Irrawaddy dolphins (*Orcaella brevirostris*) and Ganges river dolphins (*Platanista gangetica gangetica*).

	*Orcaella brevirostris*		*Platanista gangetica gangetica*	
	(N = 15)		(N = 29)	
Click parameters *	Mean ± SD	[Min; Max]	Mean ± SD	[Min; Max]
SL_pp_ (dB re 1 µPa pp at 1 m)	**194.5±3.6**	**[188.6; 199.5]**	**183.3±3.4**	**[174.8; 188.7]**
SL_RMS_ (dB re 1 µPa RMS at 1 m)	**185.1±3.6**	**[180.1; 191.2]**	**173.3±3.4**	**[164.6; 179.1]**
SL_EFD_ (dB re 1 µPa^2^ *s at 1 m)	**136.3±3.4**	**[131.1; 142]**	**126.6±3.3**	**[118.4; 132.1]**
D_-10dB_ (µs)	**13.44±3**	**[9.8; 20.8]**	**21.7±2.2**	**[16.6; 26]**
Fc (kHz)	**94.6±9.7**	**[70.2; 109]**	**61.4±4.9**	**[54]**; **[72]**
Fp (kHz)	**100.7±19.9**	**[65.2; 125]**	**58.8±6.8**	**[44.7; 73.3]**
BW −_3 dB_ (kHz)	**64.4±15.8**	**[40.2; 91.4]**	**43.8±7.1**	**[32; 62.3]**
BW −_10 dB_ (kHz)	**117.9±15.1**	**[83.9; 143.9]**	**73.2±8.7**	**[58; 98]**
BW _RMS_ (kHz)	**29.9±3.7**	**[22.3; 36.5]**	**20±2.4**	**[15.1; 25]**
Q_RMS_	**3.2±0.3**	**[2.8; 3.7]**	**3.1±0.3**	**[2.5; 3.6]**
ICI (ms)	**44.8±24.6**	**[21; 229]**	**35±18.4**	**[4.6; 125.5]**

* Click parameter abbreviations: SL_pp_ : peak-to-peak source level; SL_RMS_ : RMS source level within a −10 dB energy window; SL_EFD_: Energy flux density source level within a −10 dB energy window; D_-10dB_: Click duration (−10 dB energy window); Fc: centroid frequency; Fp: peak frequency; BW: Bandwidth (−3 dB, −10 dB or root-mean-square); Q_RMS_: Ratio of centroid frequency to RMS bandwidth; ICI: Inter-click interval.

**Table 2:** Comparative overview of biosonar parameters from other toothed whales.

	SL_pp_	SL_EFD_	D_-10dB_	Fc	BW_-3dB_	BW_-10dB_	Weight *	Reference
	dB re. 1 µPapp @1 m	dB re. 1 µPa^2^s @1 m	µs	kHz	kHz	kHz	kg	
*Physeter macrocephalus*	220–236 dB rms	195	120	15–20	*N/A*	10–15	<57000	[17], [18]
*Ziphius cavirostris*	214	164	200	42	12	23	<3000	[26]
*Hyperoodon ampullatus*	203	169	276	43	*N/A*	*N/A*	<7500	[25]
*Grampus griseus*	220	164	40	75	27	66	<400	[11]
*Pseudorca crassidens*	220	163	30	49	35	63	<2000	[11]
*Lagenorhynchus albirostris*	Up to 219	N/A	10–30	95	30	*N/A*	220–350	[48]
*Stenella attenuata*	212	150	43	83.4	*N/A*	*N/A*	<119	[49]
*Stenella longirostris*	208	148	31	80.4	*N/A*	*N/A*	<82	[49]
*Tursiops aduncus*	205	146	18	91	70.8	120.4	<270	[12], [23]
*Lagenorhynchus obscurus*	Up to 210	N/A	<70	81	67.4	N/A	<100	[22]
*Lagenorhynchus cruciger*	197	146	115	128	8	13	<94	[28]
*Phocoena* *phocoena*	192	137	79	136	16	30	45–70	[29]
*Lagenorhynchus australis*	185	133	92	129	15	*N/A*	115	[27]
*Cephalorhynchus commersonii*	177	125	78	133	21	*N/A*	<86	[27]
*Cephalorhynchus hectori*	177	121	57	128	20	30	<57	[28]
*Orcaella* *brevirostris*	195	136	13	95	64	118	115–130	This paper
*Platanista* *gangetica* *gangetica*	183	127	22	61	44	73	<75	This paper

* Values for mean or maximum recorded weights are taken from Marine Mammals of the world [81].

**Figure 5:**
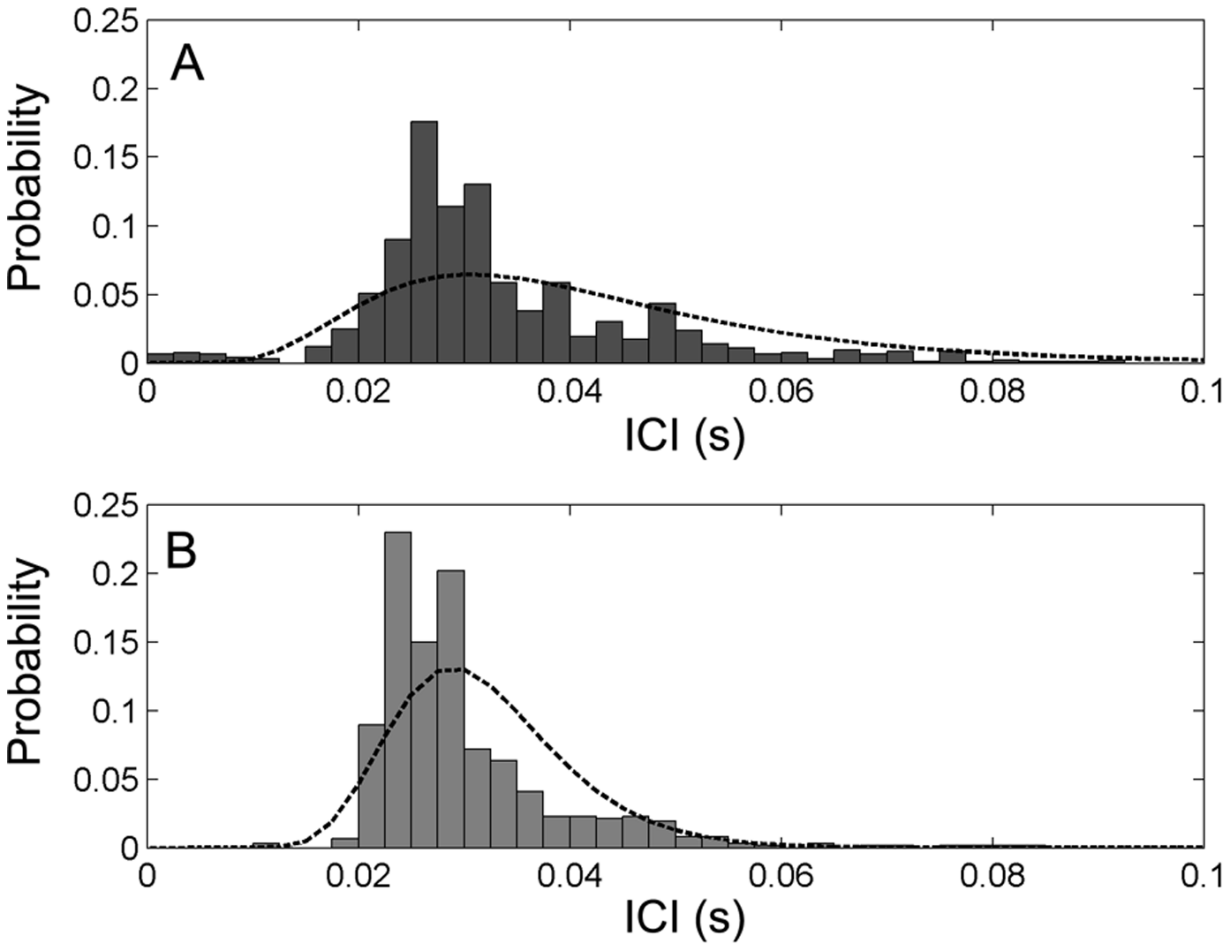
Interclick intervals of Irrawaddy dolphins and Ganges river dolphin echolocation signals. Histograms show the distribution of interclick intervals for clean series of off-axis clicks from Irrawaddy dolphins (A) and Ganges river dolphins (B). Black interrupted lines show log-normal probability density functions fitted to the data. For Irrawaddy dolphins, median ICI was 30.1 ms (N = 923) while for Ganges river dolphins, median ICI was 27.8 ms (N = 614).

Interclick intervals were measured for both species for all on-axis clicks. The ICI values for on-axis clicks were higher than ICI values measured across entire click series. Interclick intervals (mean±SD) for Irrawaddy dolphin on-axis clicks was 44.8±24.6 ms and for Ganges river dolphin on-axis clicks it was 35.0±18.4 ms (Table 1). In addition, the ICI was measured for entire click series with good signal-to-noise-ratio (SNR) and only one clicking animal at a time. A total of 923 clicks across 41 click series were analyzed for the ICI values of Irrawaddy dolphins and 614 clicks across 25 click series for Ganges river dolphins. For the entire click series, ICI (mean±SD) for Irrawaddy dolphins was 33.5±13.5 ms, and for Ganges river dolphins it was 29.9±9.0 ms (Fig. 5).

To test the potential for species discrimination in passive acoustic monitoring, probability density functions for Ganges river dolphin and Irrawaddy dolphin centroid frequencies were calculated using means and standard deviations from this paper, and assuming a normal distribution. In addition, a normalized probability density function for the Yangtze finless porpoise species (*Neophocaena phocaenoides asiaeorientialis*) was calculated using peak frequency (comparable to centroid frequency for narrowband high frequency species) and standard deviations from Li et al. [42]. An estimated best separation criterion of 72.5 kHz provided a theoretical 98.7% correct classification of Ganges river dolphin clicks and 98.9% correct classification of Irrawaddy dolphin clicks, whereas an estimated best separation criterion of 112.35 kHz provided 97.2% correct classification of Irrawaddy dolphins and 96.7% correct classification of finless porpoises. For off-axis clicks, spectral distortion increases low-frequency energy so centroid frequency estimates decrease. This meant that the classification of Irrawaddy dolphins decreased to 72.7% (N = 971) with the remainder being misclassified as Ganges river dolphins. Ganges river dolphins, in contrast, were successfully classified 99.2% of the time (N = 641).

## Discussion

The study of toothed whale biosonar signals has developed rapidly during the last decade. Most studies have focused on marine delphinids and have revealed consistent high amplitude, highly directional echolocation signals from these species (Table 2). Here, we recorded two small toothed whale species inhabiting areas that are more acoustically complex compared to the open ocean environments of many delphinids to better understand the evolutionary factors shaping different biosonar parameters of echolocating toothed whales.

Both species produce broadband echolocation clicks (Fig. 3) characterized by a short duration and a low Q ratio of centroid frequency to RMS bandwidth of around 3. A short, broadband echolocation click is characteristic of all whistling delphinids [14], [25] as well as sperm whales [17], [18]. The family platanistidae is an ancient evolutionary lineage that diverged not long after physeteridae [4], [13]. Its use of short, broadband clicks corroborates the hypothesis that the echolocation signal evolved by the shared ancestor of toothed whales was a short, broadband click that gradually evolved towards higher frequencies as greater high-frequency hearing sensitivity [43] co-evolved with the capacity for high-frequency sound production.

Echolocating toothed whales normally wait until the echo from a potential target has been received before producing a new click, meaning that the interclick interval between clicks exceeds the two-way travel time plus a processing lag time [14]. When animals are searching, the interclick interval may also reflect the limits of their environment, such as the back wall of a pool [44] or for a deep-diving animal, the altitude above the sea floor where the animal is operating [45]. The interclick interval is therefore often taken as a maximum estimate of the acoustic search range of an echolocating animal [20], [46]. The two animals studied here both had higher click repetition rates compared to Indo-Pacific bottlenose dolphins (*Tursiops aduncus*) [12] and even higher click repetition rates than coastal harbor porpoises [mean ICI: 80.5 ms, 47] and riverine Yangtze finless porpoises [mean ICI: 60.4 ms, 47]. This indicates that both Irrawaddy dolphins and Ganges river dolphins were searching for prey within a shorter range than most other studied odontocetes [47].

Concurrent with the higher repetition rates, the two species also produced echolocation signals with much lower source level compared to similar sized marine delphinids. Irrawaddy dolphins (mean source levels ± SD of 194.7±4 dB re 1 µPa pp at 1 m) and Ganges river dolphins (183.6±3.5 dB re 1 µPa pp at 1 m) echolocate at more than 10 dB to 20 dB (respectively) lower source levels than other small, oceanic delphinids such as free-ranging pygmy killer whales (*Feresa attenuata* [40]), bottlenose dolphins (*Tursiops sp.* [12]), white-beaked dolphins (*Lagenorhynchus albirostris* [48]), spinner (*Stenella longirostris*) and spotted dolphins (*Stenella attenuata*) [49], and dusky dolphins (*Lagenorhynchus obscurus*, max 210 dB pp [22]) (table 2). Common to these species is that they often forage in an environment where background noise is the limiting factor that determines how far away the faint echoes from prey organisms can be detected. In a noise-limited echolocation scenario, the echo-to-noise ratio increases proportionally with the source level so that a greater detection range can be achieved by increasing the amplitude of the outgoing signals [38]. For many of these exclusively marine species, the detection range of sparse, patchily distributed prey is a crucial parameter for survival. Selection for a long detection range would therefore promote the evolution of high-amplitude echolocation signals within the constraints provided by the size of the animal, principally the dimensions, composition and biomechanics of the sound-generating nasal structures [50].

The overall body size of many oceanic delphinids is larger than the animals studied here, and it is possible that this size difference could account for the lower source levels of our animals. Indeed, large echolocating animals tend to produce echolocation clicks at high source levels (Table 2) and scaling of source level with body size might explain the low source levels produced by small species such as dusky dolphins [22]. However, Ganges river dolphins are about the same size as dusky dolphins and spinner dolphins [5] and produce similar biosonar clicks (as characterized by short duration and low Q) but with a maximum measured source level of 191 dB re 1 uPa (pp), about 20 dB lower than the maximum measured source levels for the dusky dolphins [22]. Irrawaddy dolphins are larger than both dusky dolphins and Ganges river dolphins yet produce source levels on average nearly 10 dB lower than dusky dolphins. Porpoises and other NBHF species have also been thought particularly adapted to coastal environments, and these species are mostly similar in size or smaller than the Ganges river dolphin. The longer duration of NBHF signals compared to broadband delphinid signals means that it is most appropriate to compare the click energy flux density between species. Source levels of porpoises are comparable to the two species recorded here, with source energy flux density (SL_EFD_)for harbor porpoises (*Phocoena phocoena*) (mean SL_EFD_: 137 dB re 1 µPa^2^*s [29]) similar to the source energy flux density of Irrawaddy dolphin clicks; Peale’s dolphins (*Lagenorhynchus australis*) with somewhat intermediate source levels (mean SL_EFD_: 133 dB re 1 µPa^2^*s [27]); and Commerson’s dolphins (*Cephalorhynchus commersonii*) with source levels as low as Ganges river dolphin (mean SL_EFD_: 125 dB re 1 µPa^2^*s [27]). However, while porpoises and other NBHF species resemble the two study species here both in size and source level, they echolocate at much higher peak and centroid frequencies around 130 kHz. These species have seemingly undergone evolutionary selection for a high-pass filtered biosonar signal, possibly to avoid predation from other toothed whales such as killer whales (*Orcinus orca*) [51], [52]. Ganges river dolphins diverged out early in the evolution of *odontoceti* [13], and it is unlikely that these animals ever risked predation by killer whales. However, the NBHF signal type is a subsequently derived biosonar signal that comes at the cost of a smaller bandwidth and thereby presumably less information about the acoustic environment and it does not help explain why the two species in this study produce source levels below those of oceanic delphinids.

One important challenge that these animals face is the task of locating and catching food in an acoustic habitat with high reverberation and clutter levels. Several studies have shown how close proximity to clutter [53] or to the bottom [54] may interfere with the detection of targets. Reverberation from the bottom will necessarily depend on signal frequency, grazing angle, bottom sediment type, and especially depth [54]. The two species here both forage for sparse prey through relatively shallow environments (10–15 m in the Sundarbans [33]). While it is difficult to quantify both underwater clutter and reverberation, it is reasonable to assume that a shallow, restricted river habitat provides more challenging acoustic conditions than the open ocean. Unlike a noise-limited situation, higher source levels do not help detect targets in either reverberation or clutter limited conditions, as the backscattered echo from clutter or bottom will be just as much greater as the echo from potential targets [55]. In addition, forward masking of the outgoing click [56] may play an increasingly important role for toothed whales echolocating at very close range. Consequently, we argue that the acoustic properties of the shallow-water habitat might have favored the use of clicks with relatively low source levels in Irrawaddy and Ganges river dolphins.

If reverberation can play an important role in shaping the source levels of echolocating toothed whales, this might also explain the lower source levels found for the Indo-pacific bottlenose dolphins (*Tursiops aduncus*) in a shallow coastal habitat, compared to deep-water common bottlenose dolphins (*Tursiops truncatus*) [12]. While common bottlenose dolphins are capable of detecting a metal target on a sandy bottom at up to 70 m range despite the clutter caused by the environment [57], the typical prey of Irrawaddy dolphins and Ganges river dolphins constitute small fish and shrimp [9], [58]. The low target strength and varied bottom composition in shallow water may prove to be a more complex discrimination task for the animals than detecting high target strength, metal objects. While quantitative measurements of prey target strength and reverberation in different river habitats are needed to support this, we hypothesize that both Irrawaddy dolphins and Ganges river dolphins gain an advantage by using low source level clicks for detecting and discriminating small prey items in shallow-water, cluttered environments. This is not unknown among echolocating animals. Brinkløv et al. [59] demonstrated that the long-legged bat (*Macrophyllum macrophyllum*) gradually decreased the source levels of its echolocation calls when operating in three increasingly cluttered environments. Clutter-imposed constraints from such habitats may have resulted in microchiropteran bats having specialized into guilds inhabiting different foraging niches [34], [60], with longer detection range seemingly favored for open space foragers compared to bats hunting within dense vegetation [34]. This situation may be paralleled for source levels of toothed whales: Oceanic delphinids use high source levels to find prey at long range in open areas; Irrawaddy dolphins utilize coastal habitats and venture upriver while using intermediate source levels for echolocation; and Ganges river dolphins, which diverged early from the remaining toothed whales and evolved in a spatially restricted freshwater habitat, received little advantage from long-range echolocation and use the lowest measured source levels best suited for echolocating prey at short range. It therefore seems that the selective pressures that have favored the evolution of high frequency, high source level biosonar signals in marine toothed whales cannot be extrapolated to the complex acoustic habitats of freshwater cetaceans.

**Figure 6:**
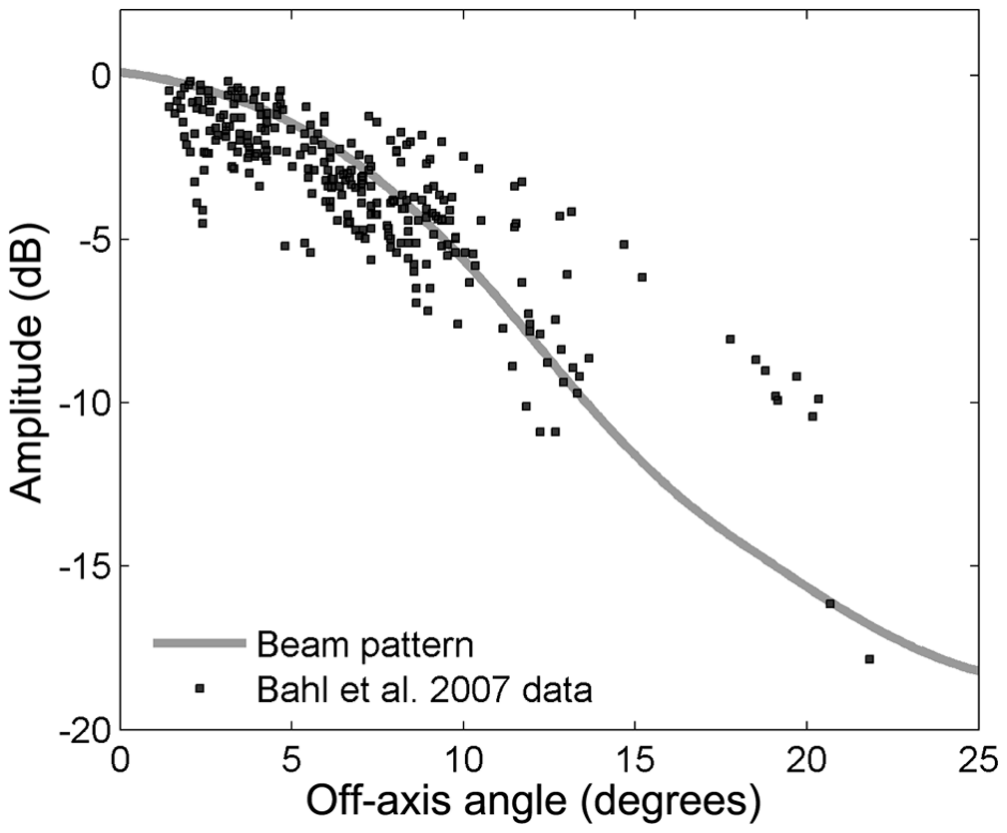
Directionality of Ganges river dolphin biosonar. Composite horizontal directionality plot of biosonar signals from Ganges river dolphins with original data (black squares) redigitized from Bahl et al. [65]. Gray line is a best fitting piston model of an on-axis click transmitted through a circular piston with a radius of 9.7 cm. The symmetrical -3 dB beamwidth of the fitted piston model is 14.5 degrees.

**Figure 7:**
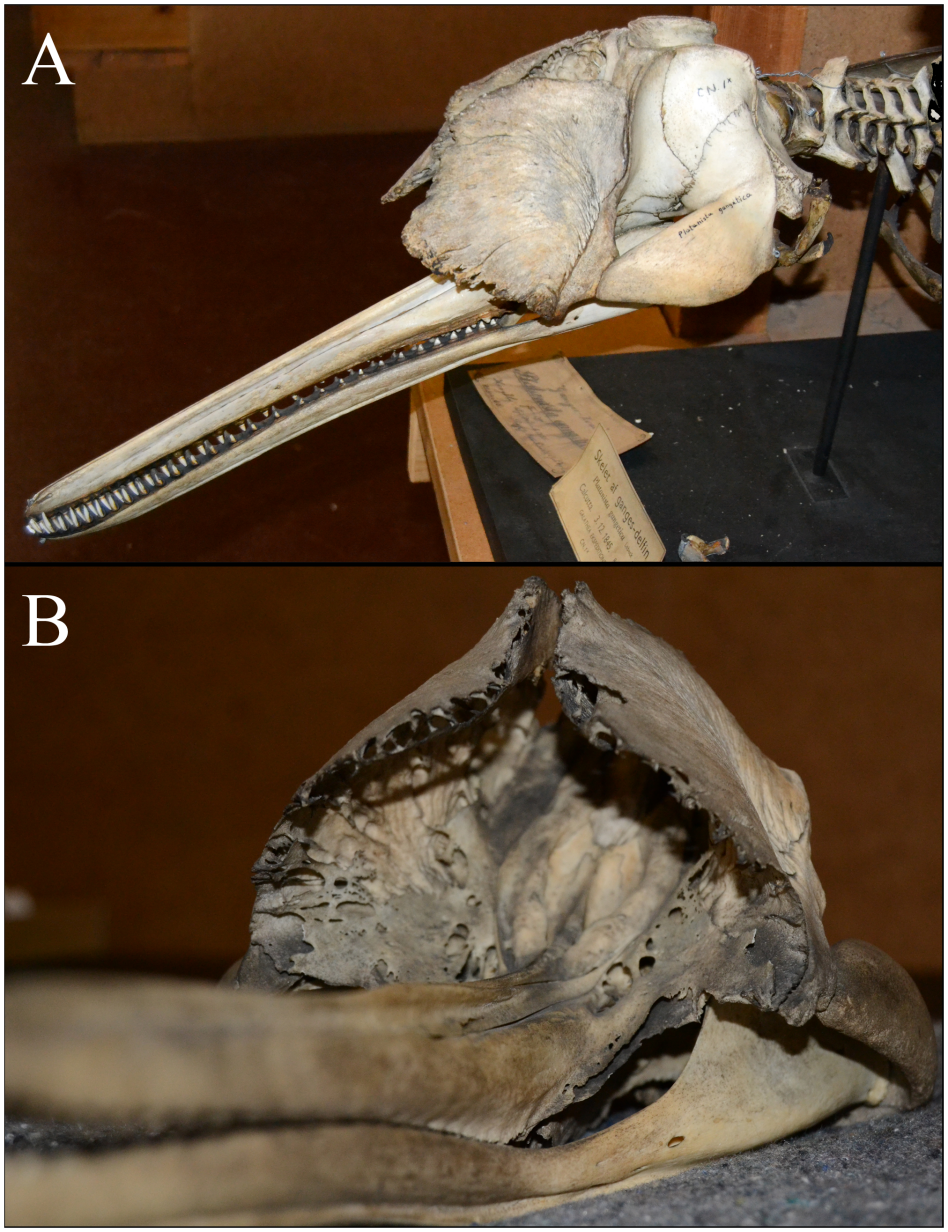
The unique cranial morphology of Ganges river dolphins. Cranial morphology of a Ganges river dolphin as seen from A) a left lateral and slightly anterior viewpoint, and B) an anterior viewpoint looking back along the anterior-posterior axis. Notice the unusual, highly porous bony maxillary crests that project anteriorly over the rostrum and nearly encircle the melon. Photos by A. Galatius.

A central component in the high source levels of toothed whales is the production of a narrow echolocation beam through partial collimation of the acoustic energy [14], [61]. Evolution appears to have favored toothed whales with a high directionality index that seems to be remarkably similar across species [15], with horizontal −3 dB (half-power) beamwidths reported between 13.1 degrees for a harbor porpoise [15] to 6.5 degrees for a beluga (*Delphinapterus leucas*) [62] and 6.2 degrees for a false killer whale (*Pseudorca crassidens*) [63]. Large odontocetes (such as sperm whales or beaked whales) can achieve a certain directionality with lower frequencies than smaller whales (such as porpoises or small delphinids) [37], [63], [64] and this might explain the overall negative correlation between biosonar frequency and body size in toothed whales (Table 2). From this relationship between body size and frequency, we would predict a relatively high centroid frequency of around 80–100 kHz for the moderately sized Irrawaddy dolphins and a higher centroid frequency of around 80–120 kHz for the small Ganges river dolphins. While Irrawaddy dolphins produced clicks with a relatively high centroid frequency (mean of 92 kHz), the Ganges river dolphins produced clicks with a surprisingly low centroid frequency (a mean±SD of 61.4±4.9 kHz) compared to their body size (Table 1). Other toothed whales of similar size use biosonar centroid frequencies of around 70–85 kHz (Pygmy killer whales) [40], 80 kHz (Hawaiian spotted dolphins and spinner dolphins [49]), 90–100 kHz (Dusky dolphins [22]) and around 130 kHz for the many NBHF species [27], [28]. The measured centroid frequency and the small size of the Ganges river dolphin would predict approximately half the directionality (6 dB smaller DI) and consequently a much broader beamwidth compared to delphinids and porpoises [14], [15]. Using equations derived from Au et al. [64] and Madsen and Wahlberg (2007), the Ganges river dolphin should have a symmetric −3 dB beamwidth of some 20 degrees and a directionality index (DI) of some 19 dB. This prediction conflicts with findings reported in the only paper investigating the directionality of Ganges river dolphins: Bahl et al. [65] reported that the −3 dB beamwidths of the Ganges river dolphins were in the order of 10 degrees in the horizontal plane and 14 degrees in the vertical plane. We find a similar, but slightly higher value, when fitting the data from Bahl et al. (2007) with a piston that best describes the variation in the data [12], [25], [27], [28]. The data indicate a single-lobed sound beam like all other toothed whales studied so far [15] rather than the peculiar, double-lobed sound beam reported in the early literature [66]. The best-fitting piston model provides a composite beamwidth of 14.5 degrees in the horizontal plane (Fig. 6). Such a half power beamwidth corresponds to a DI of 22 dB which is comparable to [64] or slightly lower than [15] the half power beamwidth of harbor porpoises, but around 3 dB (50%) better directionality index than predicted from the low frequency clicks and the small head size of the Ganges river dolphin [64]. Thus, somehow Ganges river dolphins seem to generate a beam directionality that, albeit slightly lower than most toothed whales, is comparable to that of similar sized toothed whales operating almost an octave higher in frequency. The reason for this apparent discrepancy might well lie in the unusual head anatomy of this species: Ganges river dolphins possess two unusual bony maxillary crests that project anteriorly over the facial region and virtually encircle the melon (Fig. 7). They are asymmetrical and skewed to the left, and their ventral surfaces are dominated by a thin network of air sacs that seem to have grown dorsally from the pterygoid air sinus system [67], [68]. Purves and Pilleri [69] and Pilleri and colleagues [66] proposed that the crests might function in directing the sound from the melon. It is thus possible that these air-filled bony crests could help provide a better directionality than expected from scaling, and hence explain why Ganges river dolphins can produce clicks at centroid frequencies about an octave below what should be predicted from their size and still achieve a sufficient directionality. These findings support the notion that one of the evolutionary drivers for the echolocation click frequency in toothed whales is indeed directionality. The estimated beamwidth of Ganges river dolphins is still in the broad end of measured toothed whale biosonar beams. While this might be considered a more primitive condition, a slightly wider beam combined with the greater short-range maneuverability of these animals (a consequence of having completely free cervical vertebrae [70]), may facilitate the capture of highly maneuverable prey items at close range throughout a shallow, cluttered rivers habitat.

**Figure 8:**
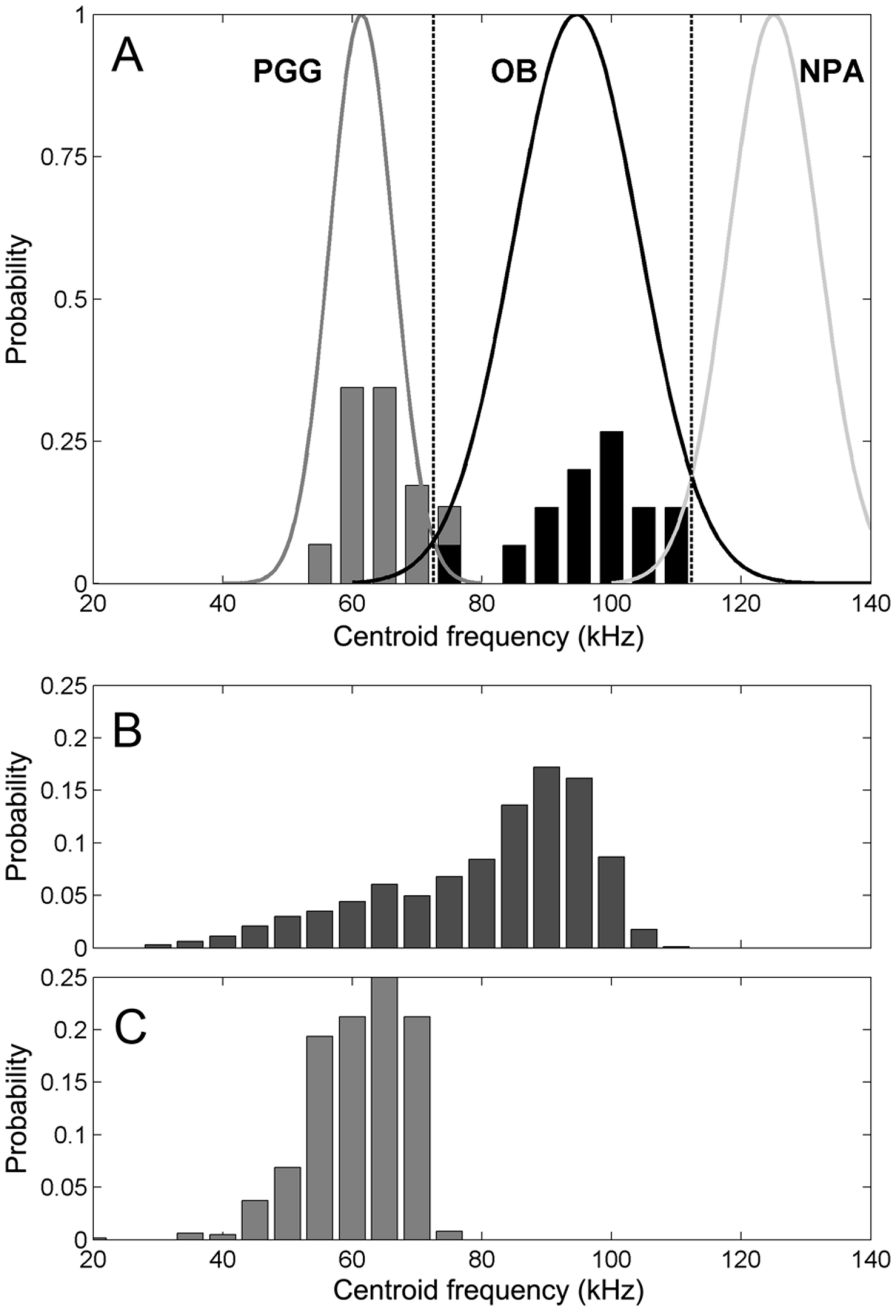
Species discrimination based on centroid frequency relevant for passive acoustic monitoring in the Sundarbans. A: Theoretical normalized probability density functions based on centroid frequency estimates (mean ± SD from Table 1) from Ganges river dolphins (grey: PGG), and Irrawaddy dolphins (black: OB) and based on peak frequency estimates from Yangtze finless porpoise (NPA) [42] assuming normally distributed estimates. Abbreviations are for latin species names. Stacked bar plot indicates probability density of centroid frequency estimates for this study. Best separation criterion (stipled lines) provides a theoretical 98.7% correct classification of Ganges river dolphin clicks and 98.9% correct classification of Irrawaddy dolphin clicks. B+C: For off-axis clicks, spectral distortion increases low-frequency energy so centroid frequency estimates decrease (B: Irrawaddy dolphins, and C: Ganges river dolphins), reducing success rate of Irrawaddy classifications to 72.7% (N = 971) with the remainder being misclassified as Ganges river dolphins, and with Ganges river dolphins being classified successfully 99.2% of the time (N = 641).

The significant difference in frequency content for these two species (Table 1) might be useful for acoustic species recognition such as seen in songbirds and other animals [71], [72], and arguably also for some sympatric delphinids [27]. Passive acoustic monitoring efforts may exploit such differences to locate critical species-specific hotspots for these endangered species [73]. The three toothed whale species typically found in the coastal and river areas of the Sundarban National Forest include *Platanista gangetica gangetica*, *Orcaella brevirostris* and *Neophocaena phocaenoides*. The on-axis biosonar centroid frequencies of these species are well separated, and spectral parameters may be a promising way of both detecting and discriminating these animals acoustically (Fig. 8). However, because biosonar signals are somewhat distorted when recorded off the acoustic axis, signals recorded away from the acoustic axis will have a lower frequency emphasis (Fig. 8 B and C). Applying the centroid frequency criteria that best separates on-axis clicks (Fig. 8) to a long series of clicks that would resemble what a passive acoustic monitor could record, results in clicks from Ganges river dolphins classified correctly nearly all the time (99.2% correct classification) whereas clicks from Irrawaddy dolphins were classified less successfully (72.7% correct classification). This results in some Irrawaddy dolphin clicks being incorrectly classified as Ganges river dolphins. The same degree of spectral distortion does not happen with NBHF clicks, whereby passive acoustic monitoring would be able to detect the presence of both finless porpoises and Irrawaddy dolphins reliably. Other criteria would be necessary to reliably classify Ganges river dolphins and discriminate such detections from off-axis Irrawaddy dolphins. One way of doing this would be to shift the separation criteria slightly upwards, and to use only the maximum centroid frequency for a series of clicks. For this dataset, reliable discrimination would be achieved based on the maximum frequency of 11–15 clicks and evaluated using a separation criterion of 74 kHz. In addition to spectral species discrimination, source levels presented here would be essential for estimating the detection function of an acoustic monitoring system, providing the basis for quantifying abundance of these threatened freshwater species [74].

Acoustic monitoring has proven to be a powerful method for determining range, seasonality, and abundance of animals [75], [76] and may prove essential for understanding the population parameters of cryptic, aquatic animals such as beaked whales [77], [78] or finless porpoises [79]. Freshwater dolphins all face significant extinction risks, primarily due to habitat loss and fisheries interactions, which led to the recent functional extinction of the Baiji (*Lipotes vexillifer*) [80]. Robust acoustic discrimination mechanisms that allows for monitoring of Irrawaddy dolphins and Ganges river dolphins could be especially helpful for managing protected areas such as the three new wildlife sanctuaries that were established by the Government of Bangladesh in the Sundarbans for the conservation of both species [73] and provide better information that can help prevent a continued decline or extinction of these two threatened freshwater species.

### Conclusion

Irrawaddy dolphins and Ganges river dolphins within the river systems of the Sundarban mangrove forest use high repetition rate, low source level echolocation clicks compared to marine species of similar size. Whereas obligate marine delphinids use high source level echolocation signals, Irrawaddy dolphins, inhabiting coastal and upriver habitats, produce lower source levels, with mean source levels of 194.7 dB (max 203 dB) re 1 µPa_pp_ and Ganges river dolphins, living exclusively in a shallow river habitat, produce even lower source levels of 183.6 dB (max 191) re 1 µPa_pp_. The ultimate cause of these low source levels may be a relaxed selection for long-range echolocation inhabiting restricted, shallow, geomorphically complex river systems, with limits on echolocation range imposed by reverberation and clutter. Interestingly, the centroid frequency of the clicks used by Ganges river dolphins is almost an octave lower than expected from their size. The unusual, air-filled bony maxillary crests found in this species may compensate in part for this lower frequency by providing a larger effective baffle and hence a more directional sound beam than the biosonar frequency and head size would predict. The beamwidth of Ganges river dolphins is still wider than most other toothed whales, and it is possible that this may facilitate capture of highly maneuverable prey items in shallow water. Acoustic discrimination between freshwater odontocetes may facilitate acoustic monitoring efforts and may help prevent a continued decline of these two threatened freshwater species.

## References

[1] Griffin DR (1958) Listening in the dark: the acoustic orientation of bats and men. New Haven CT: Yale University Press. 464 p.

[2] Schevill WE, McBride AF (1956) Evidence for echolocation by cetaceans. Deep Sea Research (1953) 3: 153–154.

[3] Jones G, Teeling EC (2006) The evolution of echolocation in bats. Tr Ecol Evol 21: 149–156. 10.1016/j.tree.2006.01.001 16701491

[4] McGowen MR, Spaulding M, Gatesy J (2009) Divergence date estimation and a comprehensive molecular tree of extant cetaceans. Mol Phylogenet Evol 53: 891–906. 19699809 10.1016/j.ympev.2009.08.018

[5] Jefferson TA, Leatherwood S, Webber MA (1994) Marine Mammals of the World. Rome: FAO. 320 p.

[6] Madsen PT, Payne R, Kristiansen NU, Wahlberg M, Kerr I, et al (2002) Sperm whale sound production studied with ultrasound time/depth-recording tags. J Exp Biol 205: 1899–1906. 12077166 10.1242/jeb.205.13.1899

[7] Watwood SL, Miller PJO, Johnson M, Madsen PT, Tyack PL (2006) Deep-diving foraging behaviour of sperm whales (*Physeter macrocephalus*). J Anim Ecol 75: 814–825. 16689963 10.1111/j.1365-2656.2006.01101.x

[8] Benoit-Bird KJ, Würsig B, Mfadden CJ (2004) Dusky dolphin (*Lagenorhynchus obscurus*) foraging in two very different habitats: Active acoustic detection of dolphins and their prey. Mar Mammal Sci 20: 215–231.

[9] Smith BD (2002) Susu and Bhulan - *Platanista gangetica gangetica* and *P. g. minor*. In: W. F. Perrin BW, J. G. M Thewissen, editor. Encyclopedia of marine mammals. San Diego: Academic Press. 1208–1213.

[10] Au WWL (2004) Echolocation signals of wild dolphins. Acoust Phys 50: 454–462.

[11] Madsen PT, Kerr I, Payne R (2004) Echolocation clicks of two free-ranging, oceanic delphinids with different food preferences: false killer whales *Pseudorca crassidens* and Risso’s dolphins *Grampus griseus* . J Exp Biol 207: 1811–1823. 15107437 10.1242/jeb.00966

[12] Wahlberg M, Jensen FH, Soto NA, Beedholm K, Bejder L, et al (2011) Source parameters of echolocation clicks from wild bottlenose dolphins (*Tursiops aduncus* and *Tursiops truncatus*). J Acoust Soc Am 130: 2263–2274. 21973382 10.1121/1.3624822

[13] Hamilton H, Caballero S, Collins AG, Brownell RL (2001) Evolution of river dolphins. Proc R Soc Lond B Biol Sci 268: 549–556. 10.1098/rspb.2000.1385 PMC1088639 11296868

[14] Au WWL (1993) The Sonar of Dolphins: New York: Springer Verlag. 277 p.

[15] Koblitz JC, Wahlberg M, Stilz P, Madsen PT, Beedholm K, et al (2012) Asymmetry and dynamics of a narrow sonar beam in an echolocating harbor porpoise. J Acoust Soc Am 131: 2315–2324. 22423726 10.1121/1.3683254

[16] Moore PW, Dankiewicz LA, Houser DS (2008) Beamwidth control and angular target detection in an echolocating bottlenose dolphin (*Tursiops truncatus*). J Acoust Soc Am 124: 3324–3332. 19045815 10.1121/1.2980453

[17] Madsen PT, Wahlberg M, Møhl B (2002) Male sperm whale (*Physeter macrocephalus*) acoustics in a high-latitude habitat: implications for echolocation and communication. Behav Ecol Sociobiol 53: 31–41.

[18] Møhl B, Wahlberg M, Madsen PT, Heerfordt A, Lund A (2003) The monopulsed nature of sperm whale clicks. J Acoust Soc Am 114: 1143–1154. 12942991 10.1121/1.1586258

[19] Madsen PT, Wilson M, Johnson M, Hanlon RT, Bocconcelli A, et al (2007) Clicking for calamari: toothed whales can echolocate squid *Loligo pealeii* . Aquat Biol 1: 141–150.

[20] Au WWL, Floyd RW, Penner RH, Murchison AE (1974) Measurement of echolocation signals of atlantic bottlenose dolphin, *Tursiops truncatus* Montagu, in open waters. J Acoust Soc Am 56: 1280–1290. 4425222 10.1121/1.1903419

[21] Au WWL, Herzing DL (2003) Echolocation signals of wild Atlantic spotted dolphin (*Stenella frontalis*). J Acoust Soc Am 113: 598–604. 12558295 10.1121/1.1518980

[22] Au WWL, Würsig B (2004) Echolocation signals of dusky dolphins (*Lagenorhynchus obscurus*) in Kaikoura, New Zealand. J Acoust Soc Am 115: 2307–2313. 15139642 10.1121/1.1690082

[23] Jensen FH, Bejder L, Wahlberg M, Madsen PT (2009) Biosonar adjustments to target range of echolocating bottlenose dolphins (*Tursiops* sp.) in the wild. J Exp Biol 212: 1078–1086. 19329740 10.1242/jeb.025619

[24] Johnson M, Madsen PT, Zimmer WMX, de Soto NA, Tyack PL (2006) Foraging Blainville’s beaked whales (*Mesoplodon densirostris*) produce distinct click types matched to different phases of echolocation. J Exp Biol 209: 5038–5050. 17142692 10.1242/jeb.02596

[25] Wahlberg M, Beedholm K, Heerfordt A, Mohl B (2011) Characteristics of biosonar signals from the northern bottlenose whale, *Hyperoodon ampullatus* . J Acoust Soc Am 130: 3077–3084. 22087935 10.1121/1.3641434

[26] Zimmer WMX, Johnson MP, Madsen PT, Tyack PL (2005) Echolocation clicks of free-ranging Cuvier’s beaked whales (*Ziphius cavirostris*). J Acoust Soc Am 117: 3919–3927. 16018493 10.1121/1.1910225

[27] Kyhn LA, Jensen FH, Beedholm K, Tougaard J, Hansen M, et al (2010) Echolocation in sympatric Peale’s dolphins (*Lagenorhynchus australis*) and Commerson’s dolphins (*Cephalorhynchus commersonii*) producing narrow-band high-frequency clicks. J Exp Biol 213: 1940–1949. 20472781 10.1242/jeb.042440

[28] Kyhn LA, Tougaard J, Jensen F, Wahlberg M, Stone G, et al (2009) Feeding at a high pitch: Source parameters of narrow band, high-frequency clicks from echolocating off-shore hourglass dolphins and coastal Hector’s dolphins. J Acoust Soc Am 125: 1783–1791. 19275335 10.1121/1.3075600

[29] Villadsgaard A, Wahlberg M, Tougaard J (2007) Echolocation signals of wild harbour porpoises, *Phocoena phocoena* . J Exp Biol 210: 56–64. 17170148 10.1242/jeb.02618

[30] Smith BD, Braulik G, Strindberg S, Ahmed B, Mansur R (2006) Abundance of Irrawaddy dolphins (*Orcaella brevirostris*) and Ganges river dolphins (*Platanista gangetica gangetica*) estimated using concurrent counts made by independent teams in waterways of the Sundarbans mangrove forest in Bangladesh. Mar Mammal Sci 22: 527–547.

[31] Smith BD, Reeves RR (2012) River Cetaceans and Habitat Change: Generalist Resilience or Specialist Vulnerability? J Mar Biol 2012: 11.

[32] Herald ES, Brownell RL, Frye FL, Morris EJ, Evans WE, et al (1969) Blind river dolphin: first side-swimming cetacean. Science 166: 1408–1410. 5350341 10.1126/science.166.3911.1408

[33] Smith BD, Braulik G, Strindberg S, Mansur R, Diyan MAA, et al (2009) Habitat selection of freshwater-dependent cetaceans and the potential effects of declining freshwater flows and sea-level rise in waterways of the Sundarbans mangrove forest, Bangladesh. Aquat Conserv 19: 209–225.

[34] Schnitzler H-U, Moss CF, Denzinger A (2003) From spatial orientation to food acquisition in echolocating bats. Tr Ecol Evol 18: 386–394.

[35] Spiesberger JL, Fristrup KM (1990) Passive localization of calling animals and sensing of their acoustic environment using acoustic tomography. Amer Nat 135: 107–153.

[36] Wahlberg M, Mohl B, Madsen PT (2001) Estimating source position accuracy of a large-aperture hydrophone array for bioacoustics. J Acoust Soc Am 109: 397–406.

[37] Madsen PT, Wahlberg M (2007) Recording and quantification of ultrasonic echolocation clicks from free-ranging toothed whales. Deep-Sea Res I 54: 1421–1444.

[38] Urick RJ (1983) Principles of underwater sound: Peninsula, Los Altos. 423 p.

[39] Madsen PT (2005) Marine mammals and noise: Problems with root mean square sound pressure levels for transients. J Acoust Soc Am 117: 3952–3957. 16018497 10.1121/1.1921508

[40] Madsen PT, Kerr I, Payne R (2004) Source parameter estimates of echolocation clicks from wild pygmy killer whales (*Feresa attenuata*) (L). J Acoust Soc Am 116: 1909–1912. 15532623 10.1121/1.1788726

[41] Møhl B, Wahlberg M, Madsen PT, Miller LA, Surlykke A (2000) Sperm whale clicks: Directionality and source level revisited. J Acoust Soc Am 107: 638–648. 10641672 10.1121/1.428329

[42] Li SH, Wang KX, Wang D, Akamatsu T (2005) Echolocation signals of the free-ranging Yangtze finless porpoise (*Neophocaena phocaenoides asiaeorientialis*). J Acoust Soc Am 117: 3288–3296. 15957795 10.1121/1.1882945

[43] Liu Y, Rossiter SJ, Han XQ, Cotton JA, Zhang SY (2010) Cetaceans on a molecular fast track to ultrasonic hearing. Curr Biol 20: 1834–1839. 20933423 10.1016/j.cub.2010.09.008

[44] Verfuss UK, Miller LA, Pilz PKD, Schnitzler HU (2009) Echolocation by two foraging harbour porpoises (Phocoena phocoena). J Exp Biol 212: 823–834. 19251999 10.1242/jeb.022137

[45] Jaquet N, Dawson S, Douglas L (2001) Vocal behavior of male sperm whales: Why do they click? J Acoust Soc Am 109: 2254–2259. 11386576 10.1121/1.1360718

[46] Kadane J, Penner R (1983) Range ambiguity and pulse interval jitter in the bottlenose dolphin. J Acoust Soc Am 74: 1059–1061. 6630720 10.1121/1.389940

[47] Akamatsu T, Teilmann J, Miller LA, Tougaard J, Dietz R, et al (2007) Comparison of echolocation behaviour between coastal and riverine porpoises. Deep-Sea Res II 54: 290–297.

[48] Rasmussen MH, Miller LA, Au WWL (2002) Source levels of clicks from free-ranging white-beaked dolphins (*Lagenorhynchus albirostris* Gray 1846) recorded in Icelandic waters. J Acoust Soc Am 111: 1122–1125. 11865816 10.1121/1.1433814

[49] Schotten M, Au WWL, Lammers MO, Aubauer R (2004) Echolocation recordings and localization of wild spinner dolphins (*Stenella longirostris*) and pantropical spotted dolphins (*S. attenuata*) using a four hydrophone array. In: Thomas JA, Moss CF, Vater MM, editors. Echolocation in Bats and Dolphins. Chicago: University of Chicago Press. 393–400.

[50] Cranford TW, Amundin M, Norris KS (1996) Functional morphology and homology in the odontocete nasal complex: Implications for sound generation. J Morphol 228: 223–285. 8622183 10.1002/(SICI)1097-4687(199606)228:3<223::AID-JMOR1>3.0.CO;2-3

[51] Morisaka T, Connor RC (2007) Predation by killer whales (*Orcinus orca*) and the evolution of whistle loss and narrow-band high frequency clicks in odontocetes. J Evol Biol 20: 1439–1458. 17584238 10.1111/j.1420-9101.2007.01336.x

[52] Andersen SH, Amundin M (1976) Possible predator-related adaptation of sound production and hearing in the Harbour porpoise. Aquat Mamm 4: 56–57.

[53] Au WWL, Turl CW (1983) Target detection in reverberation by an echolocating Atlantic bottlenose dolphin (*Tursiops truncatus*). J Acoust Soc Am 73: 1676–1681. 6863745 10.1121/1.389389

[54] Au WWL (1992) Application of the reverberation-limited form of the sonar equation to dolphin echolocation. J Acoust Soc Am 92: 1822–1826. 1401527 10.1121/1.403838

[55] Urick RJ (1962) Generalized form of the sonar equations. J Acoust Soc Am 34: 547–550.

[56] Supin AY, Nachtigall PE, Breese M (2008) Forward masking as a mechanism of automatic gain control in odontocete biosonar: A psychophysical study. J Acoust Soc Am 124: 648–656. 18647006 10.1121/1.2918544

[57] Murchison AE (1980) Detection range and range resolution of echolocating Bottlenose Porpoise (*Tursiops truncatus*). In: Busnel RG, Fish JF, editors. Animal Sonar Systems. New York: Plenum. 43–70.

[58] Stacey PJ, Leatherwood S (1997) The Irrawaddy dolphin, *Orcaella brevirostris*: a summary of current knowledge and recommendations for conservation action. In: Morton B, Perrin WF, editors. Asian Marine Biology. Hong Kong: Hong Kong University Press. 195–214.

[59] Brinklov S, Kalko EKV, Surlykke A (2010) Dynamic adjustment of biosonar intensity to habitat clutter in the bat Macrophyllum macrophyllum (Phyllostomidae). Behav Ecol Sociobiol 64: 1867–1874.

[60] Schnitzler H-U, Kalko EKV (1998) How echolocating bats search and find food. In: Kunz TH, Racey PA, editors. Bat Biology and Conservation. Washington, D.C.: Smithsonian Institution Press. 183–196.

[61] Norris KS, Harvey GW (1974) Sound transmission in the porpoise head. J Acoust Soc Am 56: 659–664. 4415842 10.1121/1.1903305

[62] Au WWL, Penner RL, Turl CW (1987) Propagation of beluga echolocation signals. J Acoust Soc Am 82: 807–813. 3655114 10.1121/1.395278

[63] Au WWL, Pawloski JL, Nachtigall PE, Blonz M, Gisner RC (1995) Echolocation signals and transmission beam pattern of a false killer whale (*Pseudorca crassidens*). J Acoust Soc Am 98: 51–59. 7608405 10.1121/1.413643

[64] Au WWL, Kastelein RA, Rippe T, Schooneman NM (1999) Transmission beam pattern and echolocation signals of a harbor porpoise (*Phocoena phocoena*). J Acoust Soc Am 106: 3699–3705. 10615708 10.1121/1.428221

[65] Bahl R, Sugimatsu H, Kojima J, Ura T, Behera S, et al. Beam pattern estimation of clicks of a free-ranging Ganges river dolphin. OCEANS 2007: 1–6.

[66] Pilleri G, Gihr M, Purves PE, Zbinden K, Kraus C (1976) On the behaviour, bioacoustics and functional morphology of the Indus River dolphin (*Platanista indi* Blyth, 1859). Investigations on Cetacea 6: 11–141.

[67] Fraser FC, Purves PE (1960) Anatomy and Function of the Cetacean Ear. Proc R Soc Lond B 152: 62–77. 13849847 10.1098/rspb.1960.0024

[68] Berta A, Sumich JL, Kovacs K (2005) Marine Mammals, Second Edition: Evolutionary Biology. San Diego: Academic Press.

[69] Purves PE, Pilleri G (1974) Observations on the ear, nose, throat and eye of *Platanista indi* . Investigations on Cetacea 5: 13–58.

[70] Reeves RR, Brownell RL (1989) Susu - *Platanista gangetica* (Roxburgh, 1801) and *Platanista minor* (Owen, 1853). In: Ridgway SH, Harrison R, editors. Handbook of marine mammals, vol 4: River dolphins and the larger toothed whales. London: Academic Press. 69–99.

[71] Brenowitz EA (1982) Long-range communication of species identity by song in the Red-winged Blackbird. Behav Ecol Sociobiol 10: 29–38.

[72] Searby A, Jouventin P (2003) Mother-lamb acoustic recognition in sheep: a frequency coding. Proc R Soc Lond B 270: 1765–1771. 10.1098/rspb.2003.2442 PMC1691449 12964977

[73] Smith BD, Diyan MAA, Mansur RM, Mansur EF, Ahmed B (2010) Identification and channel characteristics of cetacean hotspots in waterways of the eastern Sundarbans mangrove forest, Bangladesh. Oryx 44: 241–247.

[74] Kyhn LA, Tougaard J, Thomas L, Duve LR, Stenback J, et al (2012) From echolocation clicks to animal density - Acoustic sampling of harbor porpoises with static dataloggers. J Acoust Soc Am 131: 550–560. 22280616 10.1121/1.3662070

[75] Marques TA, Thomas L, Martin SW, Mellinger DK, Ward JA, et al.. (2012) Estimating animal population density using passive acoustics. Biological Reviews: doi: 10.1111/brv.12001. 10.1111/brv.12001 PMC3743169 23190144

[76] Mellinger DK, Stafford KM, Moore SE, Dziak RP, Matsumoto H (2007) An overview of fixed passive acoustic observation methods for cetaceans. Oceanography 20: 36–45.

[77] Marques TA, Thomas L, Ward J, DiMarzio N, Tyack PL (2009) Estimating cetacean population density using fixed passive acoustic sensors: An example with Blainville’s beaked whales. J Acoust Soc Am 125: 1982–1994. 19354374 10.1121/1.3089590

[78] Zimmer WMX, Harwood J, Tyack PL, Johnson MP, Madsen PT (2008) Passive acoustic detection of deep-diving beaked whales. J Acoust Soc Am 124: 2823–2832. 19045770 10.1121/1.2988277

[79] Wang K, Wang D, Akamatsu T, Li S, Xiao J (2005) A passive acoustic monitoring method applied to observation and group size estimation of finless porpoises. J Acoust Soc Am 118: 1180–1185. 16158672 10.1121/1.1945487

[80] Turvey ST, Pitman RL, Taylor BL, Barlow J, Akamatsu T, et al (2007) First human-caused extinction of a cetacean species? Biol Let 3: 537–540. 17686754 10.1098/rsbl.2007.0292 PMC2391192

[81] Jefferson TA, Webber MA, Pitman RL (2011) Marine Mammals of the World: Academic Press. 592 p.

